# Presurgical miR‐675‐5p Associates With the Inflammatory Response Following Surgery and Increases Inflammatory Gene Expression In Vitro

**DOI:** 10.1002/jcsm.70359

**Published:** 2026-08-08

**Authors:** Narmin Akhundova, Richard Paul, Benjamin Fletcher, Martin Connolly, Mark J. Griffiths, Paul R. Kemp

**Affiliations:** ^1^ Cardiovascular and Respiratory Interface Section, National Heart and Lung Institute, VPD Building Hammersmith Campus Imperial College London London UK; ^2^ Department of Intensive Care Guy's and St. Thomas' NHS Foundation Trust London UK; ^3^ Peri‐Operative Medicine, Barts Heart Centre St Bartholomew's Hospital London UK

**Keywords:** aortic surgical procedures, critical illness myopathy, epigenetics, inflammation, muscular atrophy, transcriptomics

## Abstract

**Background:**

A loss of muscle mass and strength is a marker of poor outcomes following surgery. Changes in muscle gene expression following surgery are likely to contribute to postsurgical muscle loss, but the mechanisms controlling these changes are not known. We have previously shown that miRNAs associate with muscle loss in disease, including surgery, so may contribute to changes in gene expression. We analysed the change in muscle gene expression following surgery and compared it to pre‐ and postsurgical miRNA expression. We then analysed the effect of one of these miRNAs (miR‐675‐5p) on gene expression in myoblasts in the presence and absence of an inflammatory stimulus.

**Methods:**

Differential gene expression analysis was performed on RNAseq data from rectus femoris biopsies (*n* = 18 male patients) pre‐ and postsurgery. Gene expression was compared with miRNA expression in the same samples to identify patterns of mRNA expression associated with physiology and miRNA expression. The effect of miR‐675‐5p on gene expression in the presence and absence of TNF‐*α* was determined in LHCN myoblasts by RNAseq.

**Results:**

Postsurgery muscle had increased expression of gene sets associated with TNF‐*α* signalling (2.7‐fold) and *MYC* targets (2.7‐fold). Loss of muscle mass and strength associated with *MYC* target gene sets, but inflammatory gene sets only associated with loss of strength.

Comparing miRNA expression with postsurgery gene expression showed that presurgery miR‐675‐5p expression was most closely associated with postsurgical gene expression, indicating that the presurgery phenotype is important in determining the response to surgery. Presurgery miR‐675‐5p correlated with change in gene expression in response to surgery (*β* = 1.30, *p* < 0.001), such that individuals with the highest presurgery miR‐675‐5p had the largest change in gene expression in response to surgery.

In LHCN myoblasts miR‐675‐5p increased CCL2 protein release approximately 1.5‐fold (*p* < 0.001). RNAseq showed that, in the absence of TNF‐*α*, transfection with miR‐675‐5p increased the expression of 3422 genes with greatest enrichment of the hypoxia, myogenesis and TNF‐*α* signalling via NF‐κB gene sets. In the presence of TNF‐*α*, miR‐675‐5p increased the expression of 3403 genes, and the most enriched gene sets were the same suggesting that miR‐675‐5p increases the inflammatory response. miR‐675‐5p also increased the expression of MuRF1 in the presence and absence of TNF‐*α*.

**Conclusions:**

Male patients with higher presurgical expression of miR‐675‐5p experienced a larger increase in inflammatory gene expression following surgery. MiR‐675‐5p increased inflammatory gene expression in myoblasts suggesting that it may contribute to the size of the inflammatory response.

## 1 Background

A loss of muscle mass and strength is commonly associated with chronic and acute conditions [1]. Regardless of cause, reduced muscle mass and strength are markers of reduced survival and quality of life, having a significant economic impact. Studies of muscle wasting are complicated due to the variability in the amount of muscle lost, the number of factors that can cause muscle loss (which include inactivity, inflammation and nutrition), the interrelatedness of these factors and the fact that loss of muscle mass and strength is not entirely correlated [2].

Acute sarcopenia complicates significant surgery [3] and critical illness where it is termed intensive care unit acquired weakness (ICUAW) [4]. These precipitating events are likely to lead to significant changes in gene expression that underlie the changes in muscle phenotype. Consequently, comparing the gene expression profile before and after surgery with physiological change is one way to understand the factors that are important in the acute sarcopenia response.

We have previously analysed changes in muscle mass and strength following aortic surgery to develop a model of muscle wasting [5, 6, 7, 8, 9]. In this model, we have compared the loss of muscle mass and strength with circulating GDF‐15 and metabolite levels over the 7 days following surgery with muscle loss [5]. We have also shown that muscle loss associates with presurgical quadriceps miRNA expression [6, 7, 8]. These studies have shown that the presurgical phenotype (e.g., miR‐424 and miR‐542 expression as well as mitochondrial dysfunction) associate with the loss of muscle mass following surgery even though the size of the stress response to surgery (measured as the increase in cortisol following surgery) does not. miR‐422a, −424 and −542 also associate with physical performance in COPD patients [6, 7, 8], but we have yet to determine any association of miR‐675, another miRNA associated with muscle mass and physical performance in COPD patients [10] and older individuals [11], with muscle loss following surgery.

These data led us to hypothesise that (a) changes in the gene expression profile following surgery would associate with the loss of muscle mass and strength, and (b) levels of noncoding RNAs would associate with altered gene expression and give further insight into the biology of muscle loss. We therefore analysed quadriceps gene expression before and 24 h after surgery and compared it to changes in physiology and miRNA expression. Subsequently, we analysed the effect of miR‐675‐5p on the inflammatory response in a myoblast cell line.

## 2 Methods

### 2.1 Patients

The patients included in this study are a subset from a previously reported cohort [8] of individuals undergoing elective aortic surgery at the Royal Brompton Hospital. Patients provided written informed consent, and the study was approved by the National Research Ethics Committee (07/Q0204/68). The inclusion and exclusion criteria are given in [8], the demographics, type of surgery and the clinical aspects of the surgery are given in Tables S1 and S2 with the comorbidities and drugs used after surgery given in Tables S3 and S4. Rectus femoris cross‐sectional area (RF_CSA_) was quantified by ultrasound before and 7 days after surgery and a loss of > 10%RF_CSA_ in 7 days was used to define wasting as described previously [12]. Handgrip and quadriceps strength were determined as previously described [8]. The RNAseq substudy used the nine male patients with the greatest muscle loss and the nine male patients with the least muscle loss with adequate pre‐ and postsurgery sample. To reduce variability the study was restricted to males as these were the largest group in the main study even though muscle loss was greater in females. Open biopsies of the rectus femoris were taken by the surgical team under general anaesthesia at the time of surgery and 24 h later the Bergstrom method under local anaesthesia was used as previously described [6, 13].

### 2.2 Assessment of miRNA

MicroRNA expression was analysed in Trizol extracted RNA and quantified by Taqman qRT‐PCR as described in Supporting Information.

### 2.3 Quantification of mRNAs

Individual mRNAs were quantified in Trizol extracted RNA by quantitative real time PCR (qRT‐PCR) as previously described [14], using the primers shown in Table S5. mRNA data derived from qRT‐PCR were normalised using *RPLPO* as a reference gene from the muscle or by B2 microglobulin and HPRT from cells in culture.

The transcriptome of muscle biopsies and cells was analysed by next generation sequencing performed on RNA isolated using Trizol as described in Supporting Information.

### 2.4 Bioinformatic Analysis

Analysis of the RNAseq data is described in Supporting Information. Briefly Bam files were generated in Galaxy (usegalaxy.eu [15]) and used in the R programme DESeq2 [16] to identify differentially expressed genes. TPM extracted from DESeq2 were used in WGCNA [17] to generate modules and compare transcript abundance with defined phenotypic variables. Differential gene expression and correlation coefficients were used as ranking metrics for Gene set enrichment using GSEA.

### 2.5 In Vitro Analysis

Cell culture: LHCN cells (from Prof Mouly, Université Paris Cité) were cultured in 12 well plates in skeletal muscle growth medium (PromoCell) supplemented with 20% FCS as previously described [7] for 3 days to allow them to grow to > 90% confluence. The cells were transfected with miRVana miR‐675‐5p or Control oligonucleotide using lipofectamine and treated with TNF‐*α* as described in Supporting Information.

For ELISA quantification of CCL2, cells were transfected as above. Following transfection, the cells were placed into growth medium for 18 h then transferred into differentiation medium to stop further proliferation. Cell culture medium was collected 72 h later and quantified for CCL2 by ELISA (CCL2/MCP‐1 duoset ELISA kit, R and D systems) according to the manufacturer's instructions.

### 2.6 Statistical Analysis

Statistical analysis was carried out in R (DESeq2 and WGCNA) or in Aabel 3.0 or SPSS v31, as described in Supporting Information.

## 3 Results

### 3.1 Changes in Gene Expression in Response to Surgery

DESeq2 analysis of pre‐ vs. postsurgery gene expression identified 1417 genes that increased following surgery and 1196 genes that decreased p_adj_ < 0.05 (Table S6). The five genes showing the largest fold increase were *MT1A, SAA2*, CHI3L1, *LINC01554* and *SAA1*; the five showing the largest fold decrease were the long noncoding RNAs *AC062015.1, AC093843.1* and *LINC01356* and the coding genes *CTXN3* and *CHST9* (Table S6).

Gene set enrichment using the Hallmark set showed that the genes increased were enriched for targets of the transcription factor *MYC* and inflammation, including TNF‐*α* signalling and IL6‐JAK STAT signalling, at an FDR *q* < 0.001 (Table 1). Those reduced in response to surgery did not show significant enrichment in this analysis.

**TABLE 1:** Gene set enrichment for differentially expressed genes in response to aortic surgery.

Hallmark set	Normalised Enrichment	NOM *p*‐val	FDR *q*‐val
MYC targets V2	2.70	< 0.001	< 0.001
TNFA signalling via NF‐KB	2.70	< 0.001	< 0.001
Unfolded Protein Response	2.38	< 0.001	< 0.001
MYC targets V1	2.37	< 0.001	< 0.001
Reactive oxygen species pathway	2.20	< 0.001	< 0.001
IL6 JAK STAT3 signalling	2.19	< 0.001	< 0.001
Hypoxia	2.16	< 0.001	< 0.001
P53 pathway	1.95	< 0.001	< 0.001
Inflammatory response	1.94	< 0.001	< 0.001
UV response up	1.91	< 0.001	< 0.001
IL2 STAT5 signalling	1.85	< 0.001	0.001
MTORC1 SIGNALLING	1.82	< 0.001	0.001
Oestrogen response early	1.66	< 0.001	0.006
TGF‐beta signalling	1.64	0.011	0.007
G2M checkpoint	1.52	< 0.001	0.024
E2F targets	1.39	0.007	0.060

**FIGURE 1:**
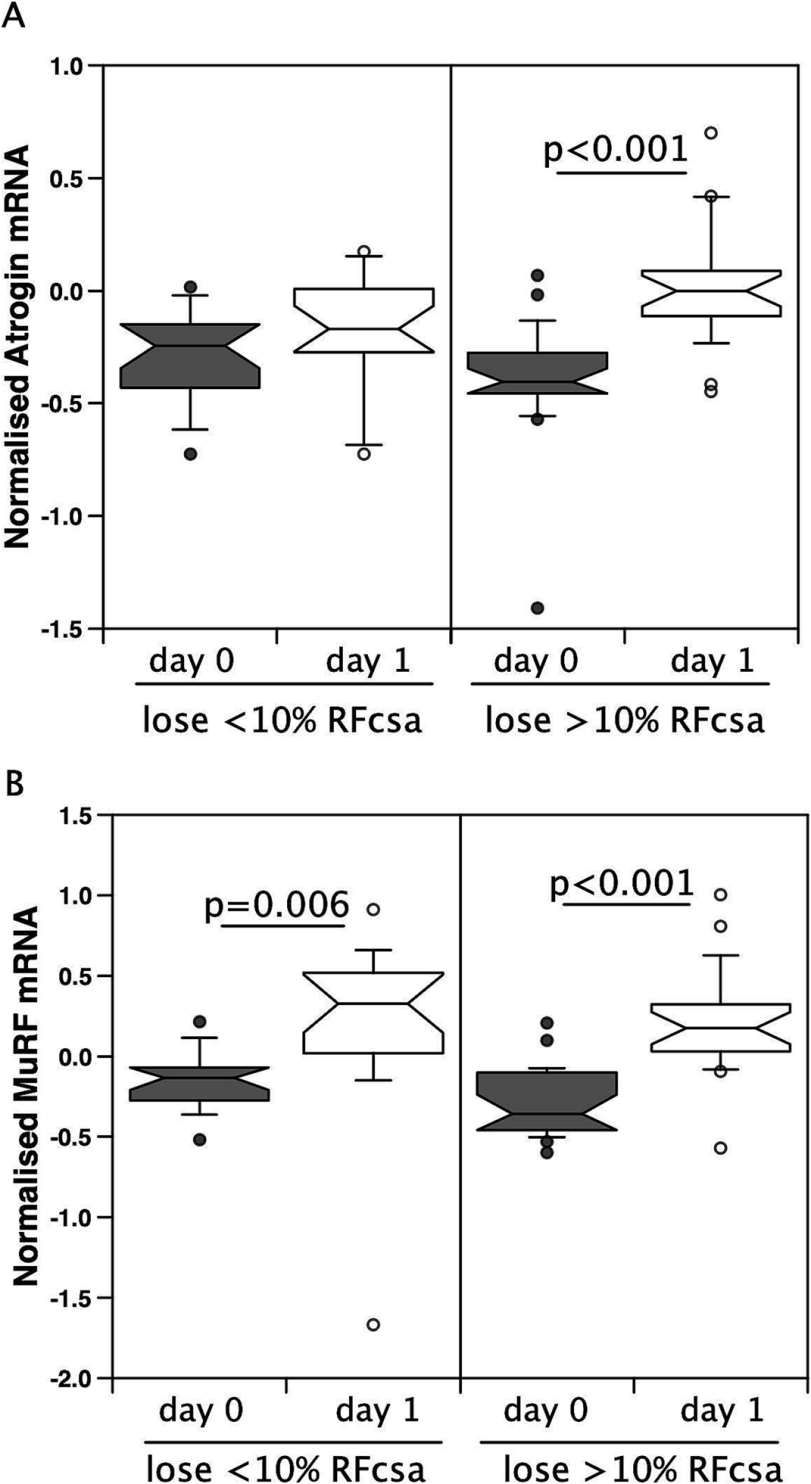
Expression of Atrogin and MuRF in response to aortic surgery: The expression of Atrogin (A) and MuRF1 (B) was quantified by qPCR in quadriceps biopsies taken following the induction of anaesthesia but before surgery (presurgery) and 24 h after surgery (postsurgery). Notched box and whisker plot (whiskers to 10th and 90th centiles + outliers) showing that in patients who lost more than 10% of the cross‐sectional area of the rectus femoris (RFcsa) both Atrogin and MuRF1 were significantly elevated postsurgery compared to presurgery (both at *p* < 0.001, ANOVA). In patients who lost less than 10% RFcsa only MuRF1 increased significantly (*p* = 0.006). *n* = 19 nonwasting and 21 wasting patients.

Comparison of the expression of the muscle ubiquitin ligases MuRF1 (*TRIM63*) and Atrogin (*FBXO32*) pre‐ and postsurgery showed that both increased 24 h after surgery (MuRF1, p_adj_ = 0.0006, Atrogin‐1, p_adj_ = 0.031). These increases were confirmed by qRT‐PCR using the complete cohort of 40 samples (Figure 1). Comparing the change in expression of the ubiquitin ligases in wasting (defined as those who lost > 10% RF_csa_ as described in [5]) and nonwasting patients (those who lost < 10% RF_csa_) separately showed that MuRF1 increased in both groups, but Atrogin‐1 only increased significantly in the wasting group (Figure 1).

We hypothesised that the postsurgery transcriptome would reflect changes in muscle mass and strength so compared the expression of all postsurgery genes with loss of muscle mass (RF_CSA_ %) and percentage loss of quadriceps or handgrip strength using biweight midcorrelation (WGCNA) and used the correlation coefficients as the ranking metric for GSEA.

### 3.2 Loss of Muscle Mass

GSEA using Hallmark showed the components of the postsurgery muscle transcriptome that most closely positively associated with loss of muscle mass were targets of the *MYC* and E2F transcription factors (Table 2A) and components of cell stress responses (unfolded protein response, DNA repair and G2M checkpoint). Gene sets most negatively associated with muscle loss included epithelial–mesenchymal transition (EMT), adipogenesis and the UV response.

**TABLE 2:** Gene set enrichment analysis of gene expression on postsurgery associating with loss of muscle mass (A) strength (B) 7 days after surgery.

(A) Postsurgery transcriptome vs. loss of RF_CSA_ (%)			
hallmark	Normalised enrichment	NOM *p*‐val	FDR *q*‐val
MYC targets V2	2.20	< 0.001	< 0.001
MYC targets V1	2.15	< 0.001	< 0.001
Unfolded protein response	1.99	< 0.001	< 0.001
E2F targets	1.95	< 0.001	< 0.001
DNA repair	1.86	< 0.001	0.001
G2M checkpoint	1.83	< 0.001	0.001
P53 pathway	1.45	0.003	0.053
Epithelial–mesenchymal transition	−2.77	< 0.001	< 0.001
Adipogenesis	−2.17	< 0.001	< 0.001
UV response DN	−1.95	< 0.001	0.001
Coagulation	−1.89	< 0.001	0.001
Cholesterol homeostasis	−1.87	< 0.001	0.001
Angiogenesis	−1.76	0.005	0.004
Notch signalling	−1.76	< 0.001	0.004
Myogenesis	−1.75	< 0.001	0.003
Interferon‐alpha response	−1.74	< 0.001	0.003
Apical junction	−1.65	< 0.001	0.007
Fatty acid metabolism	−1.57	< 0.001	0.015
Oestrogen response late	−1.53	< 0.001	0.020
Hypoxia	−1.52	0.004	0.020
Oxidative phosphorylation	−1.49	< 0.001	0.023
Oestrogen response early	−1.39	0.013	0.054
KRAS signalling up	−1.38	0.009	0.057

(B) Postsurgery transcriptome vs. loss of QMVC (%)			
Hallmark	Normalised enrichment	NOM *p*‐val	FDR *q*‐val
MYC targets V2	1.80	0.002	0.006
E2F targets	1.77	< 0.001	0.005
G2M checkpoint	1.73	< 0.001	0.006
Unfolded protein response	1.68	0.001	0.009
TNFA signalling via NF‐KB	1.52	0.003	0.051
IL6 JAK STAT3 signalling	1.51	0.022	0.046
Epithelial–mesenchymal transition	−1.99	< 0.001	0.004
Oxidative phosphorylation	−1.88	< 0.001	0.006
Myogenesis	−1.76	< 0.001	0.009
Notch signalling	−1.49	0.013	0.051

### 3.3 Loss of Strength

The Hallmark gene sets associated with the loss of quadriceps strength included several gene sets also associated with loss of muscle mass (e.g., *MYC* and E2F, and G2M checkpoint) as well as some associated with the inflammatory response (TNF‐*α*, IL6 and interferon gamma), suggesting that the loss of muscle mass and strength are regulated by overlapping but distinct gene expression profiles, with inflammatory gene sets likely to contribute more to loss of strength. The Hallmark gene sets negatively associating most strongly with loss of quadriceps strength at *q* < 0.05 were EMT (Table 2B) and oxidative phosphorylation. Loss of handgrip strength also showed positive associations with *MYC* and E2F targets, and inflammatory signalling, but also showed a negative association with genes associated with oxidative phosphorylation (Table S7). Even though the biopsies were taken from the quadriceps, so measured quadriceps gene expression patterns, the net enrichment scores between gene sets and the strength measurements tended to be stronger with loss of handgrip strength perhaps due to greater reliability of measurement. Consistent with this suggestion, in the whole cohort the association between loss of quadriceps mass and loss of handgrip strength (*r* = 0.638, *p* < 0.001) was stronger than with loss of quadriceps strength (*r* = 0.484, *p* = 0.002).

### 3.4 MicroRNAs in the Loss of Muscle Strength and Mass

**FIGURE 2:**
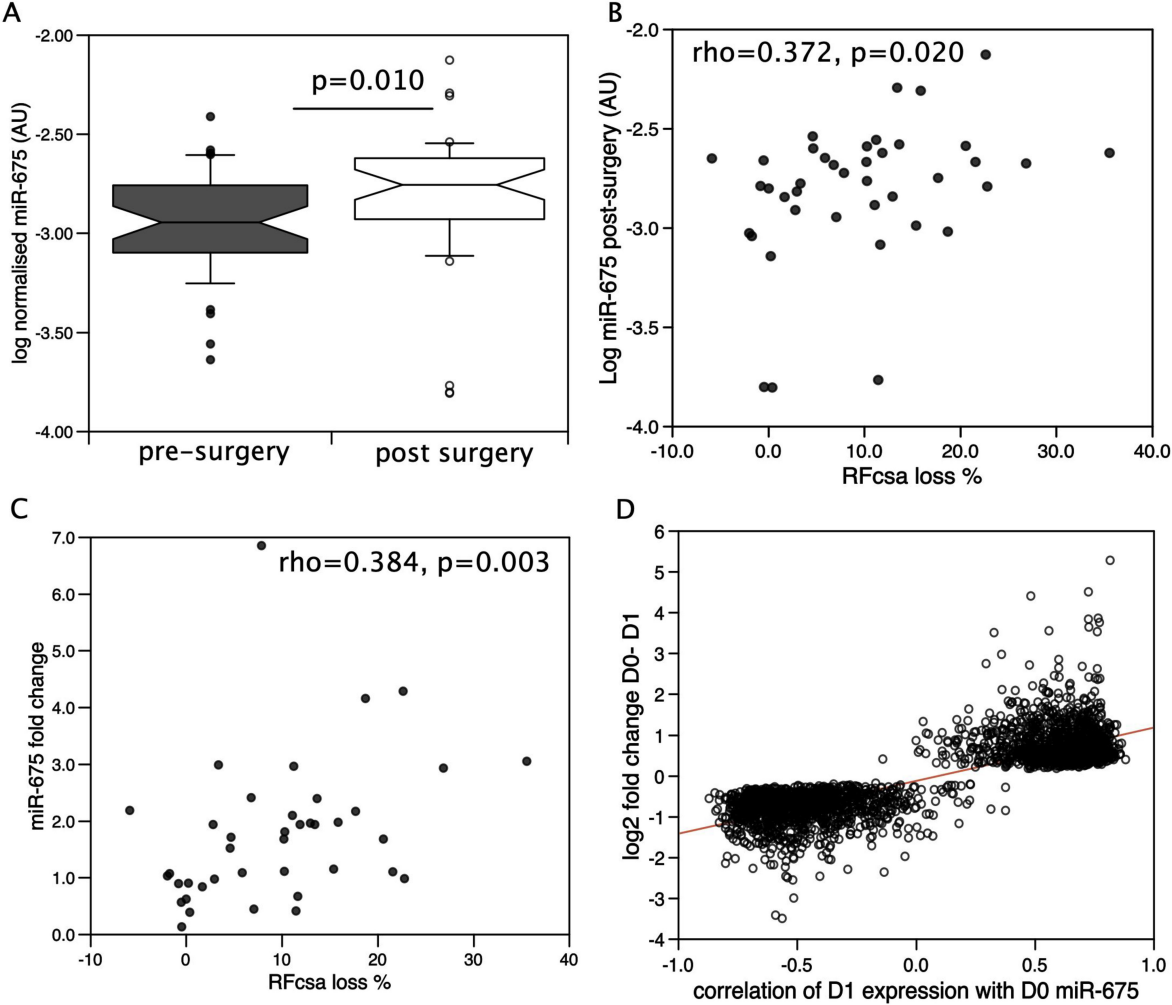
Expression of miRNAs in response to aortic surgery: The expression of miR‐675‐5p was quantified by qPCR in quadriceps biopsies taken following the induction of anaesthesia but before surgery (presurgery) and 24 h after surgery (postsurgery). (A) Comparing miR‐675‐5p expression before and after surgery showed that it increased following surgery (*p* = 0.010). (B and C) Both postsurgical miR‐675‐5p expression (B) and the fold‐change in miR‐675‐5p from pre‐ to postsurgery (C) showed positive correlations with loss of rectus femoris cross‐sectional area (RFcsa%) by Day 7 (*ρ* = 0.372 95% CI (0.06–0.62) *p* = 0.020 and *ρ* = 0.384 95% CI (0.07–0.63) *p* = 0.003, respectively *n* = 40 in both cases). Spearman's correlation was used for these analyses. (D) Comparison of quadriceps miR‐675‐5p presurgery with the change in gene expression for all genes identified as changing in response to surgery quantified by RNAseq (*n* = 18) showed that postsurgery expression of genes that increased in response to surgery was positively associated with presurgery miR‐675‐5p whereas that of genes which reduced in response to surgery were negatively associated with presurgical miR‐675‐5p expression, implying that miR‐675‐5p affected the size of change in gene expression and that individuals with higher miR‐675‐5p expression would have a larger response to surgery.

We have previously quantified miR424 and −542 [6, 7] but not miR‐675‐5p in these patients. Quantification of miR‐675‐5p in the whole cohort before and after surgery showed that it increased in response to surgery (Figure 2A) and postsurgery levels of miR‐675‐5p associated with loss of RF_csa_ 7 days later (*ρ* = 0.372, *p* = 0.020, Figure 2B). The change in miR‐675‐5p levels between pre‐ and postsurgery also associated with loss of muscle (RFcsa: *ρ* = 0.384, *p* = 0.003, Figure 2C).

To determine whether any of the miRNAs we had quantified associated with changes in gene expression, we used WGCNA to generate gene modules for the postsurgery data and then compared these modules with both the physiological parameters and the miRNA expression levels on each day. The WGCNA of the postsurgery samples identified 26 coexpression modules ranging in size from 146 to 7145 genes. Perhaps surprisingly, none of the modules showed strong associations with physiological traits, including loss of strength and muscle mass. Furthermore, neither pre‐ nor postsurgery miR‐424 (the miRNA with the strongest association with muscle loss) associated strongly with the postsurgical transcriptome modules (Figure S1), suggesting that although it affects protein synthetic capacity it contributes little to early postsurgical gene expression.

The strongest association observed was between the largest module (the turquoise module, 7145 genes) and the expression of presurgery miR‐675‐5p (*p* = 5x10^−4^, Figure S1). Indeed, this was not the only module associating with presurgery miR‐675‐5p at *p* < 0.01, as the dark grey (200 genes), blue (3174 genes) and green‐yellow (497 genes) also had associations *p* < 0.01, with four further modules at *p* < 0.05. Presurgery miR‐675‐5p was the only trait variable to show more than one module association of *p* < 0.01. These data raise the possibility that miR‐675‐5p expression at the time of surgery regulates the size of the response to surgery. Therefore, we compared presurgery miR‐675‐5p with postsurgery gene expression using biweight midcorrelation and used the correlation coefficient as the ranking metric for GSEA. This analysis showed that the genes positively associated with miR‐675‐5p were enriched for *MYC* targets and the inflammatory response (including TNF‐*α* signalling, IL6 signalling and IL2 signalling, Table 3). Gene sets negatively associated with the previous day's miR‐675‐5p included those associated with oxidative phosphorylation and myogenesis.

**TABLE 3:** Gene set enrichment for presurgery miR‐675 with the mRNAs from the muscle of aortic surgery patients postsurgery.

Hallmark	Normalised enrichment	NOM *p*‐val	FDR *q*‐val
**Positive enrichment**			
MYC targets V1	2.87	< 0.001	< 0.001
Unfolded protein response	2.62	< 0.001	< 0.001
MYC targets V2	2.62	< 0.001	< 0.001
TNFA signalling via NF‐KB	2.17	< 0.001	< 0.001
DNA repair	2.01	< 0.001	< 0.001
E2F targets	1.89	< 0.001	< 0.001
P53 pathway	1.88	< 0.001	0.001
MTORC1 signalling	1.83	< 0.001	0.001
IL6 JAK STAT3 signalling	1.81	0.001	0.001
G2M checkpoint	1.73	< 0.001	0.003
TGF‐beta signalling	1.72	0.004	0.003
Inflammatory response	1.71	< 0.001	0.003
PI3K AKT MTOR signalling	1.67	0.001	0.005
UV response up	1.60	0.002	0.010
IL2 STAT5 signalling	1.55	0.001	0.017
WNT beta‐catenin signalling	1.54	0.024	0.017
Reactive oxygen species pathway	1.47	0.034	0.034
Protein secretion	1.46	0.023	0.035
Hypoxia	1.37	0.019	0.079
**Negative enrichment**			
Oxidative phosphorylation	−2.92	< 0.001	< 0.001
Myogenesis	−2.20	< 0.001	< 0.001
Fatty acid metabolism	−1.86	< 0.001	0.003
Bile acid metabolism	−1.71	< 0.001	0.005
KRAS signalling DN	−1.42	0.019	0.046
Adipogenesis	−1.41	< 0.001	0.042

As the associations were with gene sets that change in response to surgery, we compared presurgery miR‐675‐5p expression, with the fold change for that gene in response to surgery for genes that changed at *p* < 0.05, showing that genes whose Day 1 expression positively correlated with the previous day's miR‐675‐5p expression increased, whereas those where the correlation was negative decreased. Regression analysis of this trend gave the equation [log2 fold change = 1.30*correlation coefficient −0.11] (*p* < 0.001, Figure 2D). Amongst these genes, those that associated with miR‐675‐5p at *r* > 0.6 had a median increase of 1.5‐fold, and a maximum increase of 39.1‐fold with no genes showing a reduction, whereas those that associated with miR‐675‐5p at *r* < −0.6 had a median decrease of 1.7‐fold and a maximum decrease of 4.8‐fold with no genes showing an increase. This result suggests that individuals expressing higher levels of miR‐675‐5p have a larger response to the inflammatory stimulus of surgery. Mechanistically this result could occur if miR‐675‐5p regulated the expression of regulators of the inflammatory response thereby affecting the amount of signal through inflammatory pathways.

If miR‐675‐5p was setting the size of response to surgery of inflammatory response‐ and *MYC*‐*regulated genes* in muscle, it would be predicted to associate with those same gene sets in other inflammatory conditions. We therefore compared the expression of miR‐675‐5p with gene expression previously determined by microarray in patients with chronic obstructive pulmonary disease (COPD: see Data S1 [18]). Again miR‐675‐5p was associated with inflammation and *MYC‐regulated* gene expression (Table S8).

**FIGURE 3:**
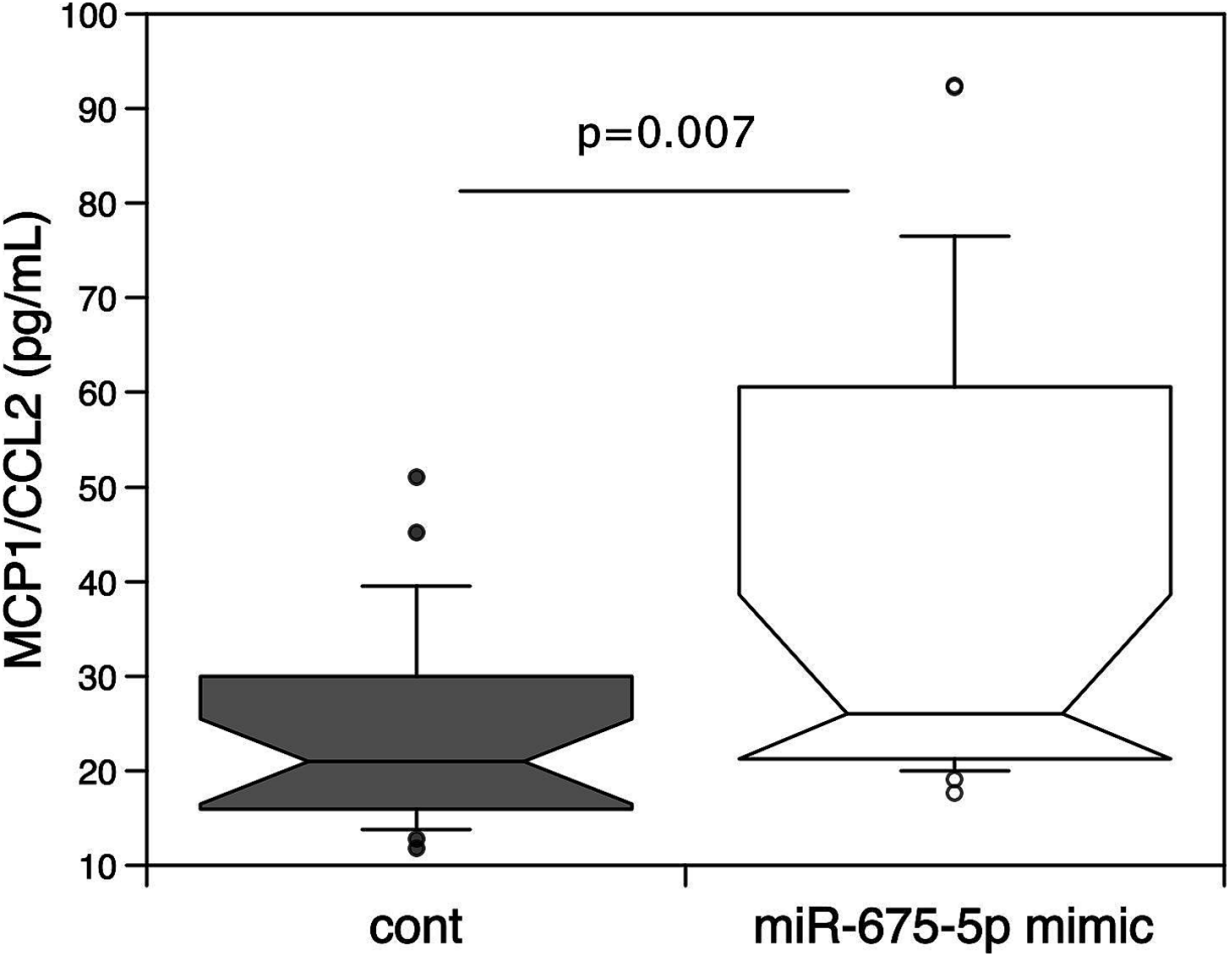
Effect of miR‐675‐5p on CCL2 production by LHCN myoblasts. LHCN cells were transfected with control oligonucleotide (cont) or miR‐675‐5p mimic (675 mim), then left overnight in growth medium before being transferred to differentiation medium for 3 days. The medium was collected and CCL2 quantified by ELISA. Figure shows a notched box and whisker plot (whiskers to 10th and 90th centiles + outliers) showing that cells transfected with miR‐675‐5p produced more CCL2 than those transfected with the control oligonucleotide. *p* < 0.001, Mann–Whitney *U* test *n* = 8 samples from each of three independent experiments.

Given that TNF‐*α* signalling via NF‐kB was a major gene set elevated in response to surgery, and associated with miR‐675‐5p following surgery and in COPD patients, we quantified CCL2 protein in the supernatant of cells transfected with miR‐675‐5p mimic or a control oligonucleotide. Transfection with the mimic increased the levels of CCL2 consistent with miR‐675‐5p increasing the expression of TNF‐*α* regulated genes (Figure 3).

To determine whether miR‐675‐5p could increase the expression of inflammatory genes, we performed RNAseq on cells transfected with miR‐675‐5p in the presence and absence of TNF‐*α*. In the absence of TNF‐*α*, miR‐675‐5p increased the expression of 3422 genes and reduced the expression of 3592 genes compared to control (Table S9). The five genes showing the greatest fold change increase were *SAA2* (serum amyloid A2 a protein involved in the innate immune response), *ADAMTS15* (a protease that contributes to muscle extracellular matrix remodelling), *MT3* (a metallothionein), *LRRC32* (a latent TGF‐beta binding cell surface molecule) and *AC004832.6* (a novel transcript). The top five genes most downregulated (fold change) by miR‐675‐5p were a readthrough gene *CRYZL2P‐SEC16B*, three noncoding RNAs *R3HDM2P1, GAPDHP72* and *TMEM246‐AS1* and the coding gene *SEC16B* (a gene involved in endoplasmic reticulum organisation). The top downregulated predicted miR‐675‐5p targets with seed sequence matches in the 3’‐UTR were *TNFRSF9* (a member of the TNF receptor superfamily) and *POST* (periostin, a matricellular protein that contributes to muscle regeneration, [19]). Three of the pseudogenes in the top 20 RNAs downregulated were *GAPDH* pseudogenes with *GAPDH* also showing significant suppression (3.8‐fold *p* = 1.22x10^−57^).

In the presence of TNF‐*α*, miR‐675‐5p increased the expression of 3402 genes and reduced the expression of 3617 genes compared to the control oligonucleotide in the presence of TNF‐*α* (Table S10). The five most elevated genes were *SOST* (sclerostin, an inhibitor of WNT signalling), an antisense gene *ST8SIA6‐AS1, ARPP21* (an RNA binding protein), *AP000550.4* (a novel transcript) and *PIP* (prolactin induced protein). The five genes most reduced were *PSG9* (an immunoglobulin superfamily member), *H2AFB1* (H2A histone family, member B1) *AC105150.1* (a novel transcript), *FHAD1* (forkhead associated phosphopeptide binding Domain 1) and *AC009318.2* (a novel transcript). Both *PSG9* and *FHAD1* contain predicted miR‐675‐5p seed sequences (MirWalk 3.0 [20]).

Analysis of the data by GSEA showed that in both cases there was positive enrichment for genes associated with hypoxia, myogenesis and inflammation, with enrichment for TNF‐*α* signalling greater than that for the other inflammatory gene sets (Table 4A,B). However, in the presence of TNF‐*α*, miR‐675‐5p increased the enrichment of inflammation gene sets to a greater extent than in its absence, implying miR‐675‐5p promotes the inflammatory response. The major gene sets showing negative enrichment were those for E2F and *MYC*. Other gene sets enriched included those associated with *KRAS* signalling and myogenesis.

**TABLE 4:** Gene set enrichment analysis of genes altered in response to miR‐675.

A. In the absence of TNF‐*α*			
Hallmark	Normalised enrichment	NOM *p*‐val	FDR *q*‐val
Positive enrichment			
Hypoxia	2.00	< 0.001	0.001
Myogenesis	1.90	< 0.001	0.001
TNFA signalling via NF‐KB	1.82	< 0.001	0.002
KRAS signalling up	1.78	< 0.001	0.003
UV response DN	1.74	< 0.001	0.004
WNT beta‐catenin signalling	1.66	0.008	0.011
Coagulation	1.64	< 0.001	0.010
Apoptosis	1.62	0.001	0.011
Inflammatory response	1.57	0.001	0.018
Oestrogen response early	1.57	< 0.001	0.016
P53 pathway	1.54	< 0.001	0.021
IL6 JAK STAT3 signalling	1.50	0.011	0.030
KRAS signalling DN	1.49	0.003	0.029
Hedgehog signalling	1.46	0.058	0.037
TGF‐beta signalling	1.45	0.034	0.039
IL2 STAT5 signalling	1.43	0.012	0.045
Negative enrichment			
E2F targets	−2.18	< 0.001	< 0.001
MYC targets V1	−1.80	< 0.001	0.002
G2M checkpoint	−1.71	< 0.001	0.008
MYC targets V2	−1.62	0.003	0.010

B. In the presence of TNF‐*α*			
Hallmark	Normalised enrichment	NOM *p*‐val	FDR *q*‐val
Positive enrichment			
Hypoxia	2.08	< 0.001	< 0.001
TNFA signalling via NF‐KB	2.07	< 0.001	< 0.001
Myogenesis	1.95	< 0.001	< 0.001
KRAS signalling up	1.90	< 0.001	< 0.001
Apoptosis	1.81	< 0.001	0.001
UV response DN	1.80	< 0.001	0.001
WNT beta‐catenin signalling	1.77	0.006	0.003
IL6 JAK STAT3 signalling	1.70	0.001	0.004
Oestrogen response early	1.64	< 0.001	0.009
KRAS signalling DN	1.63	0.001	0.008
Inflammatory response	1.63	0.003	0.008
TGF‐beta signalling	1.63	0.011	0.007
Allograft rejection	1.62	< 0.001	0.007
IL2 STAT5 signalling	1.62	0.001	0.007
Coagulation	1.59	0.004	0.008
Complement	1.54	< 0.001	0.013
Hedgehog signalling	1.45	0.047	0.036
P53 pathway	1.44	0.007	0.039
Negative enrichment			
E2F targets	−2.10	< 0.001	< 0.001
G2M checkpoint	−1.66	< 0.001	0.016
MYC targets V1	−1.61	0.000	0.016
MYC targets V2	−1.58	0.017	0.014
Bile acid metabolism	−1.49	0.007	0.026

*Note:* Cells were transfected with miR‐675 or a control oligonucleotide, left for 2 days before being serum starved for 1 h then either left in serum free medium (A) or stimulated with 10 ng/mL TNF‐*α* (B) for 4 h prior to RNA harvesting. The RNA was sequenced and differential gene expression analysed by DESeq2. NOM *p*‐val = nominal (uncorrected) *p*‐value.

Abbreviation: FDR = false discovery rate.

Previous studies have shown that miR‐675‐5p inhibits the expression of *CDC6* [21] and, consistent with that suggestion, we found a marked reduction in *CDC6* mRNA in the miR‐675‐5p transfected cells (Table S9 and S10). Interestingly, one of the most significantly elevated genes following transfection with miR‐675‐5p, both in the presence and absence of TNF‐*α*, was the activin type 2B receptor, suggesting that miR‐675‐5p would increase the sensitivity of cells to myostatin (Tables S8 and S9). Furthermore, MuRF1 (*TRIM63*) and *TRIM62*, another ubiquitin ligase associated with muscle loss [22], but not atrogin (*FBXO32*) were increased more than twofold in cells transfected with miR‐675‐5p both with and without TNF‐*α* (Tables S8 and S9). The effects of miR‐675‐5p on the expression of a subset of genes associated with inflammation were confirmed by qPCR in two further experiments. These experiments showed that miR‐675‐5p increased MuRF1 in the presence and absence of TNF‐*α* and increased expression of *CSF1, IRAK2, NFKBIE* and *TNFAIP3* that was only significant in the presence of TNF‐*α*. Furthermore, these experiments showed a reduction in *GAPDH* in both the presence and absence of TNF‐*α* as well as a reduction in *NFKB1* in the presence of TNF‐*α* (Figure S2). These observations were all consistent with the RNAseq analysis (Fig.S3).

To determine whether miR‐675‐5p increased the sensitivity of cells to an inflammatory stimulus, we used a two‐way analysis in DEseq2. In this analysis TNF‐*α* increased the expression of 224 genes and reduced the expression of 48 genes at p_adj_ < 0.05 (Table S11). The overall effect of TNF‐*α* identified by GSEA was to increase the expression of inflammatory response gene sets, and unsurprisingly, the TNF‐*α* induced gene set was the most enriched (Table S11). The combination of miR‐675‐5p with TNF‐*α* increased only one gene in comparison to the individual treatments and did not show any enhancement of the effects of TNF‐*α*, even though the inflammatory response was the most enriched gene set as this enrichment was not statistically significant.

## 4 Discussion

In this study, we analysed changes in gene expression following aortic surgery and their association with two relevant miRNAs to identify gene expression systems that contribute to acute sarcopenia. Increased expression of genes associated with inflammation and *MYC* signalling, and reduced expression of genes associated with mitochondria and oxidative phosphorylation predominated.

Loss of muscle size associated with increased expression of *MYC* targets and reduced expression of genes associated with mitochondrial function, but no strong association with the increase in inflammatory gene sets was seen. The latter do, however, associate with the loss of strength that occurs over the subsequent 7 days. Together these data suggest that although there is similarity in the gene expression profiles promoting loss of muscle size and muscle strength, there are also differences. Although miR‐424 associated with muscle loss in this model [6] and has been strongly linked to the muscle transcriptome in patients recovering from critical care [23], it did not show strong associations with postsurgical gene expression modules identified by WGCNA. This difference is likely due to the timing of the measurements and the patient population (24 h postsurgery vs. 7 days after ICU discharge) [23].

Conversely, presurgical levels of miR‐675‐5p associated with the size of the gene expression response to surgery. Consistent with this observation, transfection of cells with miR‐675‐5p increased expression of genes associated with inflammatory responses both in the presence and absence of TNF‐*α*. These data therefore support our previously published model explaining the range of muscle loss in response to disease [2], with the revision that miR‐675‐5p may promote the inflammatory response in muscle in addition to regulating myoblast proliferation.

The data suggest an important role for the transcription factor *MYC* in the control of muscle mass and strength and in the response of the muscle to surgery. *MYC*‐regulated gene sets increased following surgery and showed the strongest positive enrichment in association with loss of muscle mass and strength. We have previously shown in both men and women with COPD, that patients with more severe disease and a lower fat free mass index (FFMI) had higher expression of the same gene sets [24]. *MYC* contributes to many aspects of cell phenotype, including proliferation, differentiation, metabolism and apoptosis [25]. The contribution of these gene sets to muscle protein turnover is difficult to determine. The genes within the *MYC*‐regulated gene sets most tightly associated with loss of muscle mass in this study are a complex mixture, including genes increasing proteosome activity (e.g., *PSMB2* and *PSMC6*), which may promote muscle loss, and genes associated with the cell cycle (e.g., *XRCC6* and *CDC45*), which may contribute to regeneration. In skeletal muscle as myofibre nuclei are post mitotic, consequential changes in cell cycle gene expression would have to occur in satellite cells, myoblasts or fibroblasts and would most likely contribute to regeneration not atrophy. Thus, the observation that *MYC* gene regulation associates with loss of muscle mass suggests the former effect predominates or that the changes in expression also occur in non‐proliferating cells so do not contribute to regeneration. These data are consistent with our previous observation in patients with COPD which showed that the expression of *MYC* in muscle associated with inflammatory gene sets as well as correlating with muscle expression of *CCL2*, IL‐6 and receptors potentially involved in muscle loss (*OSMR* and *IL31RA*) [24]. The potential contribution of *MYC* to muscle loss raises the possibility of repurposing *MYC* inhibitors [26, 27] to suppress muscle wasting particularly in inflammatory conditions. However, give *MYC's* importance to proliferation and regeneration the net systemic effect of *MYC* inhibition may be detrimental. Such a therapeutic approach would need to be selective for differentiated myofibres. The expression of H19, the host gene for miR‐675, is downregulated in most tissues shortly after birth but maintained in skeletal muscle [28]. Thus, targeting miR‐675‐5p may be more beneficial in mitigating sarcopenia, especially that associated with chronic inflammatory diseases. H19 also sequesters let‐7 miRNAs that target *MYC* expression and suppress inflammation [29]. Consequently, targeting H19/miR‐675 is likely to be more selective. Notably, metformin suppresses H19 expression thereby increasing the anti‐inflammatory activity of let‐7 miRNA [29].

It is perhaps not surprising that *MYC* genes are increased in this inflammatory response as *MYC* is stabilised and activated by IKK‐*α* [30]. *MYC* increases IL6 expression in aortic aneurysm [31], and in some cancers, increased *MYC* expression promotes local macrophage recruitment. One possibility is that increased *MYC* target gene expression promotes scavenging of muscle as breakdown is promoted following surgery. Such scavenging is an important contributor to the initiation of muscle regeneration.

*MYC* regulated gene sets were suppressed, not increased, following miR‐675‐5p transfection, suggesting that this response is separate to the direct effects of miR‐675‐5p or that its antiproliferative effects and consequent effects on *MYC* activity predominate in this model. There may, however, be an indirect association between *MYC* and miR‐675‐5p via H19, as *MYC* and H19 form a feedback loop where *MYC* promotes H19 expression and H19 promotes *MYC* expression by sequestering the let‐7 miRNAs [32].

miR‐675‐5p was associated with muscle mass and strength in COPD patients and inhibited myoblast proliferation [10]. We had therefore previously proposed that miR‐675‐5p contributed to muscle loss by limiting regeneration. The data presented here support miR‐675‐5p inhibiting cell proliferation as we confirmed that miR‐675‐5p suppressed CDC6 expression [21]. The data also show that miR‐675‐5p overexpression can increase inflammatory gene expression, suggesting that it may contribute to the inflammatory response of muscle to surgery. Although miR‐675‐5p inhibits myoblast proliferation and promotes myogenesis, it is noteworthy that the miR‐675‐5p we measured came from whole muscle. Because miR‐675‐5p increases during differentiation and the proportion of myoblast nuclei is low, it is more likely that the associations we observed—increased inflammation and muscle loss—are due to its expression in mature muscle, not in myoblasts. One alternative possibility is that miR‐675 suppresses SMAD1/5 with a consequent reduction in BMP signalling [11, 21]. In differentiated myofibres following surgery this would likely be due to reducing BMP‐stimulated protein synthesis [33, 34]. Our data do not address this issue as these SMADs have been identified as miR‐675‐3p targets [21], whereas we have focussed on miR‐675‐5p.

**FIGURE 4:**
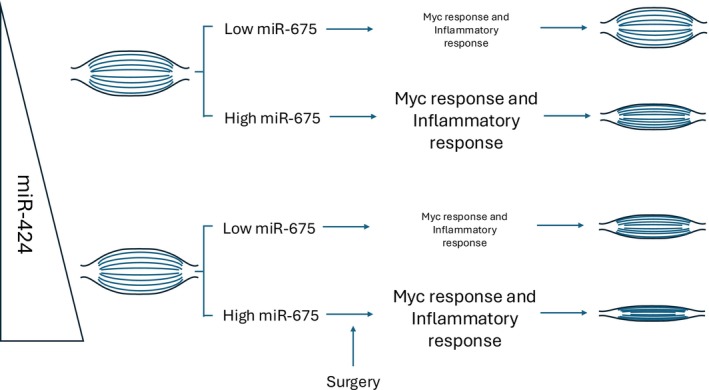
Schematic of the proposed effect of the presurgery epigenetic phenotype on muscle loss. Patients have a preexisting susceptibility to muscle wasting based on their epigenetic profile. Patients with high levels of miR‐424 are most susceptible to the loss of muscle mass in response to surgery due to reduced ribosomal [6] and translation initiation factor [35] gene expression, which will limit the ability to recover following an insult. In response to surgery there is an increase in the expression of *MYC* target‐ and inflammatory response genes, which promotes muscle loss. The size of this response is controlled by presurgical levels of miR‐675. Consequently, patients with the highest expression of miR‐424 and miR‐675‐5p are most susceptible to muscle wasting.

Although miR‐675‐5p associates with the size of change in gene expression, miR‐424 had a stronger association with muscle loss following surgery probably as a consequence of miR‐424 reducing muscle protein synthetic capacity, thus limiting recovery. Consequently, we propose that both miRNAs contribute to a presurgical phenotype that contributes to the loss of muscle in response to surgery (Figure 4). Patients with high levels of miR‐424 before surgery lose most muscle, as miR‐424 limits the amount of protein that can be synthesised by suppressing expression of essential ribosomal components [6] and translation initiation factors [35], reducing the number and synthetic capacity of ribosomes. miR‐675‐5p associates with the gene expression changes that arise after surgery. As inflammation promotes atrophy, we propose that individuals with high miR‐675‐5p may have higher expression of atrophic genes and, consistent with this suggestion, miR‐675‐5p transfection increased MuRF1 and *TRIM62* RNA.

### 4.1 Limitations of the Study

This study examines changes in quadriceps gene expression in patients undergoing aortic surgery and associates these with changes in muscle mass and strength. The data are therefore observational and do not prove causation.

The association of miR‐675‐5p with inflammatory signalling and the strength of the response to surgery are also correlative in the patients but are supported by the in vitro analysis, which shows that increasing miR‐675‐5p in a cell of muscle origin increases the expression of genes associated with inflammation both in the presence and absence of TNF‐*α* stimulation. Given that a major mechanism by which cells respond to many inflammatory cytokines is through the activation of transcription factors (e.g., NF‐kB and Jak–STAT pathways), these changes in gene expression profile suggest that miR‐675‐5p alters the response of cells to inflammatory stimuli. However, as we analysed the effects of miR‐675‐5p in myoblasts rather than muscle fibres it is possible that similar effects do not occur in differentiated muscle. Alternatively, it could be argued that the observation of the effects in a different system add strength to the potential role of miR‐675‐5p in regulating inflammation in a broader context and are consistent with numerous studies linking H19 (the miR‐675‐5p host gene) to inflammation [36, 37, 38]. It is possible that, as miR‐675‐5p promotes differentiation by reducing cell proliferation and targeting the BMP signalling system [21], the increased expression of inflammation associated genes we observe in vitro is a consequence of differences in gene expression profiles between differentiated and undifferentiated muscle cells. However, although myogenesis is identified as an enriched gene set in the presence of miR‐675, the cells were maintained in growth medium limiting the capacity for fusion, and there was no effect of miR‐675‐5p on skeletal muscle myosin heavy chain expression. Furthermore, any differences in differentiation are unlikely to explain the associations observed in the human muscle studies. Conversely, a role in the regulation of inflammation offers a plausible explanation for the association of miR‐675‐5p with muscle loss in COPD [10] and those observed here. Nevertheless, these observations need to be confirmed in an animal model where the miRNA can be expressed in fully differentiated muscle and without altering differentiation or proliferation.

The ability of miR‐675‐5p to increase the expression of genes associated with inflammation is of interest as it suggests that this miRNA is a potential target to suppress the inflammatory response in patients. It is therefore interesting to note that COPD patients with a reduced FFMI have higher miR‐675‐5p than those with a normal FFMI, and a similar association has been observed in older individuals [10, 11]. Furthermore, in these patients the expression of miR‐675‐5p correlates with the expression of the same gene sets as it does in response to surgery.

A further limitation of the study is that we have not investigated which genes/proteins are the targets of miR‐675‐5p that regulate the inflammatory response, so cannot define a mechanism. Given that miRNAs are generally thought to reduce mRNA stability by initially suppressing translation followed by removal of the poly‐A tail and removal of the 5′ cap [39], it is likely that the mRNA for the important regulator(s) will be suppressed by transfection with the miRNA. Several factors complicate identification of targets. The first is that, in our experiment, transfection with miR‐675‐5p suppressed the expression of 3592 genes of which 1330 contained seed sequence matches according to miRWalk 3.0 and 295 potential targets according to Targetscan. Selecting individual potential targets is therefore fraught. The second is that in addition to the suppression of many genes a similarly large number were significantly elevated including many receptors and signalling components that contribute to inflammation. This raises the possibility that part of the function of this miRNA is to modify the expression of factors that control transcription or DNA accessibility. These factors are unlikely to have inflammation as a tag in any of the databases precluding identifying them via a bioinformatic approach. Identification of the mechanism by which miR‐675‐5p increases the response to inflammation therefore will require a practical approach.

Further significant limitations of the study include the relatively small number of patients that the RNAseq analysis used and the restriction of this group to males. The rationale for this restriction was both practical (more males underwent this surgery during the study, 31 males vs. 10 females) and to reduce the likely variability in the dataset given the well‐known differences in muscle mass and fibre‐type between men and women. Our conclusions therefore must be limited to men and further studies carried out to determine whether the same changes and associations occur in women.

## 5 Conclusion

In conclusion, our data show that the size of the inflammatory response in muscle associates with presurgical expression of miR‐675 and that increased miR‐675 in myoblasts increases the expression of genes associated with inflammation. These observations raise the possibility that targeting this miRNA may suppress the acute sarcopenic response to surgery in susceptible patients.

## Funding

This work was supported by the PhD studentships from the Azerbaijan Ministry of Science and Education to support NA, the NIHR BRU at the Royal Brompton Hospital to support RP, Imperial College NHLI to support BF and the British Heart Foundation to support MC.

## Ethics Statement

All participants gave their written informed consent prior to inclusion in the study and ethical approval was obtained from the National Research Ethics Committee (approval 07/Q0204/68). The study was carried out in accordance with the ethical standards laid down in the 1964 Declaration of Helsinki and its later amendments.

## Conflicts of Interest

P.R.K. reports personal fees from GSK outside the submitted work. M.J.G. reports grants, personal fees and nonfinancial support from GSK, personal fees from BI, Silence Therapeutics and Cell catapult all outside the submitted work: All other authors have no conflicts of interest.

## Supporting information


**Data S1:** Supporting information.

**Data S2:** Supporting information.

**Table S1:** Demographics for the RNAseq cohort.
**Table S2:** Demographics for the whole cohort.
**Table S3:** Presurgery comorbidities.
**Table S4:** Postsurgical drug treatment.
**Table S5:** Primers used in this study.
**Table S6:** Differential gene expression for postsurgery vs. presurgery in response to aortic surgery. See excel sheet.
**Table S7:** Gene set enrichment for the postsurgery transcriptome with loss of handgrip strength.
**Table S8:** GSEA analysis of the COPD patient transcriptome with miR‐675‐5p.
**Table S9:** Genes differentially expressed in presence of miR‐675 and the absence of TNF‐*α*. See excel sheet.
**Table S10:** Genes differentially expressed in presence of miR‐675 and the presence of TNF‐*α*. See excel sheet.
**Table S11:** Comparison of genes differentially expressed in response to TNF‐*α* and the presence or absence of miR‐675. See excel sheet.

**Figure S1:** Biweight correlations of modules with physiological traits and miRNA expression. Modules of gene expression produced by WGCNA using postsurgery RNAseq data (*n* = 18 patients) were correlated (biweight midcorrelation) with physiological traits and with miRNA expression. The strongest associations seen were with presurgery miR‐675‐5p (Lg.675.D0). More intense brown colour represents greater positive module trait correlation and more intense blue colour representing greater negative correlation. CSA.D0: rectus femoris Cross‐sectional area presurgery RF.csa.loss..: Loss of rectus femoris cross‐sectional area by postsurgery Day 7 (%) HG.D0: Handgrip strength presurgery HG … loss: Loss of handgrip strength by Day 7 postsurgery ($) QMVC … loss: Loss of quadriceps maximum voluntary contraction by Day 7 postsurgery (%) AICU.LOS: length of stay in the ICU Hospital length of stay: length of stay in hospital postsurgery Lg***.D0: log concentration of miR‐*** presurgery Lg***.D1: log concentration of miR‐*** postsurgery.

**Figure S2:** Q‐RT‐PCR analysis of the effect of miR‐675‐5p mimic on the expression of a subset of genes identified as modified in the RNAseq dataset in the presence and absence of TNF‐*α*. LHCN cells were transfected with miR‐675‐5p mimic (675‐5p) or control (cont) oligo nucleotide and cultured for 2 days before being serum starved for 1 h then treated with 10‐ng/mL TNF‐*α* as described in methods. RNA was quantified by Q‐RT‐PCR. Data are presented as notched box and whiskers plots with whiskers to 10th and 90th centiles and outliers shown. Eight genes were selected from the genes showing a significant change in response to miR‐675‐5p or TNF‐*α* from the RNAseq data as either within the top 20 most significantly changed in response to TNF‐α (*NFKB1, NFKB2, NFKBIE, IRAK2, CSF1* and *TNFAIP3*), a potential target of miR‐675‐5p (*GAPDH*) or of biological interest in muscle (MuRF1). TNF‐*α* increased the expression of *NFKB1* (AB), NFKB2 (B), CSF1 (C), IRAK2 (D) NFKBIE (E) and TNFAIP3 (G). miR‐675 suppressed GAPDH (F) and elevated the expression of MuRF‐1 (H) in both the presence and absence of TNF‐*α*, suppressed the expression of NFKB1 (A) in the presence of TNF‐*α* and elevated the expression of CSF1 (C), IRAK2 (D) NFKBIE (E) and TNFAIP3 (G) in the presence of TNF‐*α*. Significance was analysed by two‐way ANOVA with Bonferroni–Dunn correction for individual comparisons. Data are taken from two experiments with four replicates in each experiment.

**Figure S3:** Effect of miR‐675‐5p mimic on the expression of genes showing the most significant response to TNF‐*α* in the presence and absence of TNF‐*α* LHCN cells were transfected with miR‐675‐5p mimic (675‐5p) or control (cont) oligo nucleotide and cultured for 2 days before being serum starved for 1 h then treated with 10‐ng/mL TNF‐α as described in methods. RNA was quantified by RNAseq. Eight genes are shown as described in Figure S2. Genes elevated in the presence of TNF‐*α* (NFKB1 (A), NFKB2 (B), CSF1 (C), IRAK2(D), NFKBIE (E) and TNFAIP3 (G)), a potential target of miR‐675‐5p (GAPDH, F) or of biological interest in muscle (MuRF1, H). RNAseq was performed on four separate samples derived from a single experiment.
