# Supplementary material for: Presurgical miR‐675‐5p Associates With the Inflammatory Response Following Surgery and Increases Inflammatory Gene Expression In Vitro
Source: J Cachexia Sarcopenia Muscle. 2026 Aug 8;17(4):e70359. doi: 10.1002/jcsm.70359 (PMC13451965; doi:10.1002/jcsm.70359)
Supplement: Supplementary file 1 [file JCSM-17-e70359-s004.pdf]

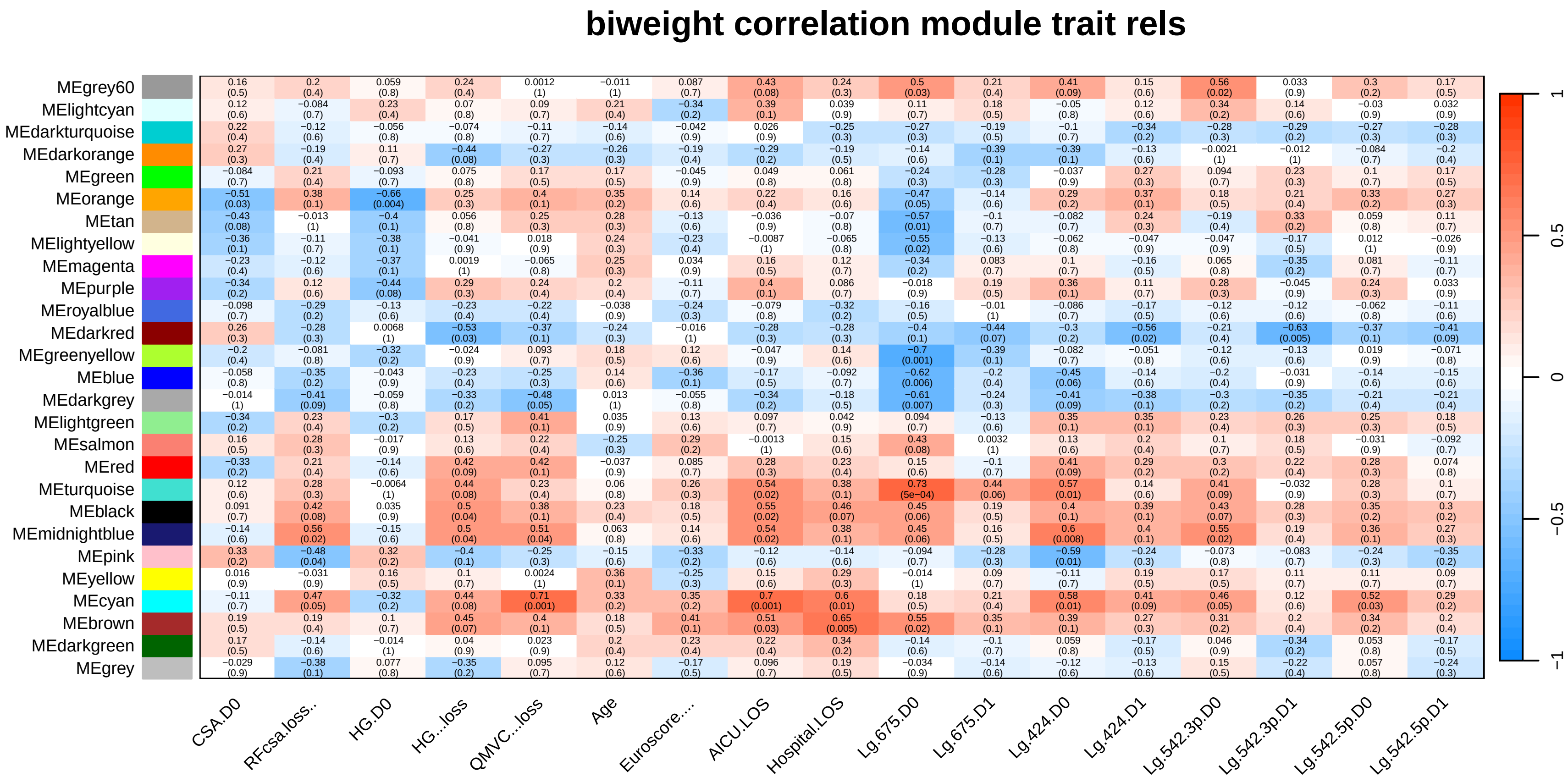
biweight correlation module trait rels
MEgrey60
MElightcyan
MEdarkturquoise
MEdarkorange
MEgreen
MEorange
MEtan
MElightyellow
MEmagenta
MEpurple
MEroyalblue
MEdarkred
MEgreenyellow
MEblue
MEdarkgrey
MElightgreen
MEsalmon
MEred
MEturquoise
MEblack
MEmidnightblue
MEpink
MEyellow
MEcyan
MEbrown
MEdarkgreen
MEgrey
CSA.D0
RFcsa.loss..
HG.D0
HG...loss
QMVC...loss
Age
Euroscore....
AICU.LOS
Hospital.LOS
Lg.675.D0
Lg.675.D1
Lg.424.D0
Lg.424.D1
Lg.542.3p.D0
Lg.542.3p.D1
Lg.542.5p.D0
Lg.542.5p.D1
1
0.5
0
−0.5
−1
