# Supplementary material for: Presurgical miR‐675‐5p Associates With the Inflammatory Response Following Surgery and Increases Inflammatory Gene Expression In Vitro
Source: J Cachexia Sarcopenia Muscle. 2026 Aug 8;17(4):e70359. doi: 10.1002/jcsm.70359 (PMC13451965; doi:10.1002/jcsm.70359)
Supplement: Supplementary file 2 [file JCSM-17-e70359-s005.docx]

**Supplementary Methods:**

**miRNA quantification**

Trizol extracted RNA isolated from muscle was reverse transcribed using MultiScribe™ Reverse Transcriptase with Megaplex™ RT Primers (human pools A and B, Version 3.0, Applied Biosystems), according to the manufacturer’s instructions. Individual microRNAs were quantified using the appropriate Taqman primers and probes (Applied Biosystems). Each reaction was performed in duplicate and the average Ct value normalised to the corresponding geometric mean of U6 and RNU48 using the ΔΔ Ct method.

**Next generation sequencing**

Next generation sequencing was performed on RNA isolated from muscle by the Genome Centre at Queen Mary University of London: data are available as GSE298105. These libraries were prepared from 100ng RNA using NEBNext® Ultra™ II Directional RNA Library Prep Kit with the NEBNext® Poly(A) mRNA Magnetic Isolation Module according to the manufacturer’s instructions. Sequencing of RNA from transfected cells was performed by the MRC Genomics facility Laboratory of Medical Sciences and are available as GSE299700. These libraries were prepared using NEBNext® rRNA Depletion Kit v2 (Human/Mouse/Rat) followed by NEBNext Ultra II Directional RNA Library Prep Kit for Illumina.

**Bioinformatic analysis:**

Individual Fastq files from each sample were loaded into the Galaxy server usegalaxy.eu[1], concatenated using ‘concatenate dataset’ trimmed using ‘trimmomatic’, aligned to hg38 using HISAT2 for paired end stranded reads with all other options set as default. Quality control was performed using Falco and Qualimap BamQC and showed a mapping efficiency >91% and a general error rate less than 0.03% for each sample.

*Deseq2:* The .bam files were loaded into the R program Deseq2[2] and the output was filtered to remove genes with low counts (by removing genes with less than 90 counts across all samples leaving 24214 genes, i.e. an average of 10 counts in 50% of the samples). A paired analysis was then carried out using patient and day as the selected variables. The data were also independently filtered as part of the default DESeq2 program. Adjusted p values (p_adj_) were calculated in DESeq2 using the Benjamani-Hochberg False discovery rate.

For the in vitro analysis, the same process was followed but filtering was carried out by setting the total number of counts at 20 leaving a total of 25621 and 27717 transcripts for analysis in the absence or presence of TNF-α respectively.

*Weighted Gene Co-expression Network Analysis (WGCNA):* The WGCNA R package[3] was used to identify clusters of genes exhibiting similar expression patterns (referred to as modules), to compare the association of these modules with physiological and biochemical parameters and to identify individual genes that correlated with these parameters irrespective of module membership.

The RNAseq data as transcripts per million (TPM) were converted to log2 (x+1) as suggested in the WGCNA package. Modules were generated separately from the pre-surgery and post-surgery data sets, using a soft thresholding-power of 8 for pre-surgery and of 7 for post-surgery, at which a scale free topology was approximated. The minimum cluster size was 100. Robust biweight mid-correlations were used for all correlation analyses. Modules were correlated with physiological parameters and miRNA expression using bi-weight mid correlation. As this component was exploratory no corrections for multiple testing were applied. Subsequently traits of interest were compared against the whole transcriptome using bi-weight mid correlation to provide a ranked list for gene set enrichment analysis.

*Gene set enrichment analysis:* Differential gene expression from DESeq2 and the biweight mid-correlation coefficients from WGCNA were used as the ranking metrics in Gene Set Enrichment Analysis (GSEA) to identify pathways associated with the physiological parameter. The ranked gene sets were compared with the Hallmark and reactome gene lists using the standard parameters in the GSEA program.

**Cell culture and transfection**

The cells were transfected using lipofectamine 2000 using 25nM oligonucleotide (miRVana miR-675-5p or Control oligonucleotide) and 2.5μL lipofectamine 2000 in 120μL OPTI-MEM for 4.5h according to the manufacturer’s instructions. After transfection the cells were transferred into growth medium for a further 2 days before either being harvested for RNA or serum starved for 1h then treated with TNF-α (10ng/mL) or control medium for 4h before being harvested for RNA. RNA was analysed by qRT-PCR. RNAseq was performed on 4 samples from each condition (control oligonucleotide transfected no TNF-α, miR-675-5p mimic transfected no TNF-α, control oligonucleotide transfected + TNF-α and mir-675-5p mimic transfected + TNF-α). Differential gene expression in the RNAseq data was determined using DESeq2.

**Statistical analysis**

Bioinformatic analysis was carried out using the Galaxy server to generate read numbers which were loaded into DEseq2 and WGCNA using R as described above. All other statistical analysis was performed using Aabel 3.0 or SPSS v31(Fisher’s exact test). Differences between two groups were quantified by Mann-Whitney U test for data sets that were not normally distributed and by t-Test for normally distributed data. Multiple groups were analysed by one way or two way ANOVA as required. Correlation analysis in WGCNA was performed using robust-biweight-mid correlation. Other correlations were carried out using Pearson’s correlation (for linear associations with correlation given as (r) or as Spearman’s correlations for non-linear correlations with the correlation coefficient given as ρ or rho. P<0.05 was taken as an indicator of statistical significance. Fisher’s exact test was used for contingency table analysis.

**COPD study**: miRNA in muscle biopsies from patients with COPD (n=41 and healthy controls, n=12) was quantified by PCR as described in Methods. These patients have previously been described and the comparison of miR-675 with fat free mass index published [4]. Microarray analysis of RNA that included the same cohort of patients was also published[5]. The demographics of the patients is described in [4] and all patients gave written informed consent to participate in the study *and the protocol was approved by the Royal Brompton & Harefield NHS Trust Research Ethics Committee (Studies 06/Q0404/35 and 06/Q0410/54).*

**Supplementary results**

To determine whether the expression of miR-675 associated with inflammatory and MYC regulated genes in a chronic inflammatory condition (COPD) we compared the expression of miR-675 which we had previously quantified [4] with gene expression determined by microarray that we had also previously published[5]. Bi-weight mid correlation was used to compare gene expression with the expression of the miRNAs. This analysis showed that genes ranked by correlation coefficient with miR-675-5p were enriched for genes regulated by MYC, and those associated with the EMT and with inflammatory signalling pathways (TNF-α-, IL2- and IL6-signalling) Table S8. Among the genes from the MYC regulated gene set list that were most positively associated with miR-675-5p were CCT2, CCT3 and NME1 which have all been shown to contribute to inflammation and inflammatory pathway activity including the regulation of NF-kB signalling and JAK-STAT signalling. These genes also show strong positive associations in the comparison of miR-675 pre-surgery with gene expression 24h post-surgery consistent with the suggestion that miR-675 is contributing to their expression in response to an inflammatory stimulus. Whether this is through the regulation of MYC activity or a separate mechanism remains to be determined.

**Supplementary references**

1.	Afgan E, Baker D, van den Beek M, Blankenberg D, Bouvier D, Cech M, et al. The Galaxy platform for accessible, reproducible and collaborative biomedical analyses: 2016 update. Nucleic Acids Res. 2016;44(W1):W3-W10.

2.	Love MI, Huber W, Anders S. Moderated estimation of fold change and dispersion for RNA-seq data with DESeq2. Genome Biol. 2014;15(12):550.

3.	Langfelder P, Horvath S. WGCNA: an R package for weighted correlation network analysis. BMC Bioinformatics. 2008;9:559.

4.	Lewis A, Lee JY, Donaldson AV, Natanek SA, Vaidyanathan S, Man WD, et al. Increased expression of H19/miR-675 is associated with a low fat-free mass index in patients with COPD. J Cachexia Sarcopenia Muscle. 2016;7(3):330-44.

5.	Willis-Owen SAG, Thompson A, Kemp PR, Polkey MI, Cookson W, Moffatt MF, et al. COPD is accompanied by co-ordinated transcriptional perturbation in the quadriceps affecting the mitochondria and extracellular matrix. Sci Rep. 2018;8(1):12165.
