# Supplementary material for: Presurgical miR‐675‐5p Associates With the Inflammatory Response Following Surgery and Increases Inflammatory Gene Expression In Vitro
Source: J Cachexia Sarcopenia Muscle. 2026 Aug 8;17(4):e70359. doi: 10.1002/jcsm.70359 (PMC13451965; doi:10.1002/jcsm.70359)
Supplement: Supplementary file 3 [file JCSM-17-e70359-s006.docx]

**Table S1 Demographics for the RNAseq cohort**

|  | **Non-wasting Patients (n=9)** | **Wasting Patients (n=9)** | **P-value** |
| --- | --- | --- | --- |
| **Demographic data** | | | |
| Age (yr) | 67 (49, 75) | 73 (67, 75) | NS |
| BMI (kg/m2) | 26.3 (26.0, 28.7) | 27.2 (24.9, 27.9) | NS |
| EuroSCORE 2 | 1.5 (1, 2.7) | 3.5 (1.7, 5.5) | NS |
| LVEF(%) | 62 (66, 45) | 55 (60, 45) | NS |
| **Operative data** | | | |
| Total bypass time (mins) | 124±39 | 131±33 | NS |
| Total cross-clamp time (mins) | 109 (62, 116) | 92 (69, 104) | NS |
| **Critical care data** | | | |
| ICU length of stay, days | 1 (1,1) | 3 (2, 7) | * |
| Hospital length of stay, days | 7.0 (6.5, 9.5) | 11.0 (9.0, 23.0) | * |
| Mechanical ventilation (hours) | 17 (14, 24) | 26 (24, 99.5) | * |
| Vasopressor duration (hours) | 37 (17, 52) | 46 (22, 114) | NS |
| Aortic and aortic valve | 4 | 1 | NS |
| aortic surgery | 2 | 3 | NS |
| aortic valve | 3 | 5 | NS |
| **Muscle data** | | | |
| RF_CSA_ day 0 | 7.4±2.2 | 6.3±0.8 | NS |
| RF_CSA_ day 7 | 7.68 (5.98, 9.14) | 5.3 (5.0, 5.6) | NS |
| Loss of RF_CSA_ (%) | 1.66 (0.54, 2.95) | 15.4 (12.9, 17.7) | ******* |
| Loss of QMVC at 7 days (%) | 0.40±9.0 | 17.9±13.1 | ****** |
| SPPB | 12 (12,12) | 12 (11,12) | NS |

BMI (Body mass index), LVEF (left ventricular ejection fraction), RFcsa (rectus femoris cross sectional area). * p<0.05, ** p<0.01, ***p<0.001. Data are presented as mean ±SD for normally distributed data or as median (interquartile range) for data that were not normally distributed

**Table S2 Demographics for the whole cohort**

|  | Non-wasting Patients (n=19) | Wasting Patients (n=21) | P-value |
| --- | --- | --- | --- |
| **Demographic data** | | | |
| Age (yr) | 58.7 ± 15.4 | 68.1 ± 14.9 | ***** |
| Sex (M/F) | 17/4 | 17/7 |  |
| BMI (kg/m2) | 27.1 ± 3.6 | 27.5 ± 6.9 | NS |
| EuroSCORE 2 | 2.0 (1.3-3.5) | 3.0 (1.4-9.6) | NS |
| LVEF(%) | 62.0 (55.5-65.0) | 57.0 (50.0, 65.0) | NS |
| **Operative data** | | | |
| Total bypass time (mins) | 143.0 ± 49.0 | 142.1 ± 55.7 | NS |
| Total cross-clamp time (mins) | 103.6 ± 35.4 | 95.7 ± 33.2 | NS |
| Aortic and aortic valve | 8 | 2 | * |
| aortic surgery | 3 | 7 | NS |
| aortic valve | 8 | 12 | NS |
| **Critical care data** | | | |
| ICU length of stay, days | 1.0 (1-2) | 3.0 (2-7) | ******* |
| Hospital length of stay, days | 8 (7-12) | 12 (9-23) | ***** |
| Mechanical ventilation (hours) | 16 (13-24) | 26 (19-99) | ****** |
| Vasopressor duration (hours) | 27 (15-48) | 46 (22-186) | ***** |
| **Muscle data** | | | |
| RF_CSA_ day 0 | 6.7 ± 2.2 | 6.1 ± 1.6 | NS |
| RF_CSA_ day 7 | 6.6 ± 2.2 | 5.2 ± 2.6 | ***** |
| Loss of RF_CSA_ in 7 days (%) | 2.2 (-0.5, 5.3) | 13.6(11.4, 20.6) | ******* |
| Loss of QMVC in 7 days(%) | 4.3 ± 10.1 | 15.0 ± 14.3 | ***** |
| SPPB | 12 (12,12) | 12 (11,12) | * |

BMI (Body mass index), LVEF (left ventricular ejection fraction), RFcsa (rectus femoris cross sectional area), SPPB (short physical performance battery). * p<0.05, ** p<0.01, ***p<0.001. Data are presented as mean ±SD for normally distributed data or as median (interquartile range) for data that were not normally distributed.

**Table S3 Pre-surgery comorbidities**

| Condition | Non-wasting patients (n=20) | Wasting patients (n= 21) | p value | Non-wasting patients (n=9) | Wasting patients (n= 9) | p value |
| --- | --- | --- | --- | --- | --- | --- |
| Ischaemic Heart Disease | 5 | 9 | NS | 2 | 4 | NS |
| Heart Failure | 2 | 3 | NS | 0 | 1 | NS |
| Re do operation | 2 | 2 | NS | 1 | 2 | NS |
| Structural Heart Disease | 16 | 17 | NS | 7 | 8 | NS |
| Aortic disease | 11 | 10 | NS | 6 | 4 | NS |
| Dysrhythmia | 5 | 10 | NS | 3 | 5 | NS |
| Hypertension | 12 | 17 | NS | 5 | 8 | NS |
| Pulmonary Hypertension | 6 | 8 | NS | 3 | 1 | NS |
| Hypercholesterolaemia | 8 | 14 | NS | 4 | 7 | NS |
| Congenital Heart Disease | 3 | 2 | NS | 1 | 1 | NS |
| Diabetes Melitus | 2 | 3 | NS | 0 | 2 | NS |
| Chronic Kidney Disease | 12 | 13 | NS | 5 | 6 | NS |
| COPD | 2 | 5 | NS | 1 | 3 | NS |

Four of the patients in the whole cohort having combined aortic and aortic valve surgery were Marfan’s syndrome patients undergoing Bentell’s procedure. However, none of these patients were included in the RNAseq cohort.

P values were calculated using Fisher’s exact test for dichotomous variables and Mann Whitney U test for continuous variables

**Table S4 Post-surgical drug treatment**

| drug | Non-wasting patients (n=19) | Wasting patients (n= 21) | p value | Non-wasting patients (n=9) | Wasting patients (n= 9) | p value |
| --- | --- | --- | --- | --- | --- | --- |
| Steroids | 2 | 4 | NS | 0 | 2 | NS |
| Noradrenaline (max dose, μg/kg/min) | 0.06 (0.04, 0.1) | 0.19 (0.07, 0.21) | NS† | 0.06 (0.05, 0.14) | 0.17 (0.07, 0.22) | NS† |
| Noradrenaline (hrs) | 25 (15, 43)† | 44 (21-114) | **† | 37 (17, 52) | 46 (22-114) | NS† |
| NMBAs | 1 | 3 | NS | 0 | 2 | NS |
| AVP | 0 | 2 | NS | 0 | 2 | NS |
| Inotropes | 3 | 10 | 0.041 | 1 | 5 | 0.050 |
| hyperglycaemia | 5 | 9 | NS | 0 | 4 | NS |
| Insulin | 5 | 9 | NS | 0 | 4 | NS |

NMBAs = neuromuscular blocking agents, AVP = arginine vasopressin. P values were calculated using Fisher’s exact test for dichotomous variables and by Mann-Whitney for continuous variables. Continuous variables give and median (interquartile range)

† 2 values missing for the non-waster data set.

**Table S5 Primers used in this study**

| **qPCR primers** | | |
| --- | --- | --- |
| **Gene** | **forward** | **Reverse** |
| Human sequences | | |
| B2MG | CTATCCAGCGTACTCCAAAG | GAAAGACCAGTCCTTGCTGA |
| RPLPO | CAGCCTCTGCATGATGTTCAGT | TCTACAACCCTGAAGTGCTTGATATC |
| MuRF1 | CCTGAGAGCCATTGACTTTGG | CTTCCCTTCTGTGGACTCTTCCT |
| Atrogin | GCAGCTGAACAACATTCAGATCAC | CAGCCTCTGCATGATGTTCAGT |
| CSF1 | CCTGCTGTTGTTGGTCTGTC | ACTGTTCCTGGTCTACAAACTCA |
| IRAK2 | ATGGCCTGCTACATCTACCA | CCGCTCCATGGACTTGATCT |
| GAPDH | GGTGGTCTCCTCTGACTTCAACA | GTTGCTGTAGCCAAATTCGTTGT |
| NFKB1 | GGATTTCGTTTCCGTTATGT | TGTCCTTGGGTCCAGCAGTT |
| NFKB2 | TGGAACCCAAGGAGCCAG | GGATAGGTCTTTCGGCCCTT |
| NFKBIE | TCACCTACACCCTGTCCTTG | TTTGAATGTCCAGGACCTCC |
| TNFAIP3 | CATGCACCGATACACACTGG | GAGAAGTGGCATGCATGAGG |

Primers are given the 5’ to 3’ direction

**Table S6 Differential gene expression for post -surgery vs pre-surgery in response to aortic surgery**

**See excel sheet**

**Table S7 Gene set enrichment for the post-surgery transcriptome with loss of handgrip strength**

| **HALLMARK** | **Normalised enrichment** | **NOM p-val** | **FDR q-val** |
| --- | --- | --- | --- |
| **Positive enrichment** | | | |
| MYC TARGETS V2 | 2.12 | <0.001 | <0.001 |
| UNFOLDED PROTEIN RESPONSE | 2.08 | <0.001 | <0.001 |
| MYC TARGETS V1 | 2.08 | <0.001 | <0.001 |
| E2F TARGETS | 2.05 | <0.001 | <0.001 |
| G2M CHECKPOINT | 1.92 | <0.001 | 0.001 |
| DNA REPAIR | 1.74 | <0.001 | 0.003 |
| TNFA SIGNALING VIA NFKB | 1.66 | <0.001 | 0.005 |
| IL6 JAK STAT3 SIGNALING | 1.62 | 0.007 | 0.008 |
| MITOTIC SPINDLE | 1.56 | <0.001 | 0.014 |
| **Negative enrichment** | | | |
| OXIDATIVE PHOSPHORYLATION | -2.86 | <0.001 | <0.001 |
| ADIPOGENESIS | -2.31 | <0.001 | <0.001 |
| EPITHELIAL MESENCHYMAL TRANSITION | -2.09 | <0.001 | 0.003 |
| MYOGENESIS | -2.08 | <0.001 | 0.002 |
| FATTY ACID METABOLISM | -1.79 | <0.001 | 0.008 |
| COAGULATION | -1.76 | <0.001 | 0.007 |
| BILE ACID METABOLISM | -1.52 | 0.031 | 0.036 |

**Table S8 GSEA analysis of the COPD patient transcriptome with miR-675-5p**

| **HALLMARK** | **Normalised enrichment** | **NOM p-val** | **FDR q-val** |
| --- | --- | --- | --- |
| EPITHELIAL MESENCHYMAL TRANSITION | 2.75 | 0.000 | 0.000 |
| MYC TARGETS V1 | 2.40 | 0.000 | 0.000 |
| ANGIOGENESIS | 2.18 | 0.000 | 0.000 |
| MTORC1 SIGNALING | 2.05 | 0.000 | 0.000 |
| UNFOLDED PROTEIN RESPONSE | 2.01 | 0.000 | 0.000 |
| ANDROGEN RESPONSE | 2.01 | 0.000 | 0.000 |
| MYC TARGETS V2 | 1.96 | 0.000 | 0.000 |
| APOPTOSIS | 1.94 | 0.000 | 0.000 |
| TNFA SIGNALING VIA NFKB | 1.80 | 0.000 | 0.002 |
| COAGULATION | 1.79 | 0.002 | 0.002 |
| XENOBIOTIC METABOLISM | 1.72 | 0.000 | 0.004 |
| CHOLESTEROL HOMEOSTASIS | 1.72 | 0.000 | 0.004 |
| P53 PATHWAY | 1.71 | 0.000 | 0.004 |
| UV RESPONSE UP | 1.68 | 0.000 | 0.004 |
| MYOGENESIS | 1.66 | 0.000 | 0.005 |
| PROTEIN SECRETION | 1.65 | 0.005 | 0.005 |
| PEROXISOME | 1.62 | 0.000 | 0.007 |
| E2F TARGETS | 1.61 | 0.002 | 0.007 |
| IL2 STAT5 SIGNALING | 1.61 | 0.000 | 0.007 |
| TGF BETA SIGNALING | 1.58 | 0.005 | 0.009 |
| REACTIVE OXYGEN SPECIES PATHWAY | 1.49 | 0.029 | 0.022 |
| APICAL SURFACE | 1.45 | 0.042 | 0.030 |
| DNA REPAIR | 1.44 | 0.005 | 0.033 |
| COMPLEMENT | 1.43 | 0.013 | 0.038 |
| KRAS SIGNALING UP | 1.42 | 0.008 | 0.038 |
| IL6 JAK STAT3 SIGNALING | 1.40 | 0.032 | 0.045 |

**Table S9 Genes differentially expressed in presence of miR-675 and the absence of TNF-α**

**See excel sheet**

**Table S10 Genes differentially expressed in presence of miR-675 and the presence of TNF-α**

**See excel sheet**

**Table S11 Comparison of genes differentially expressed in response to TNF-α and the presence or absence of miR-675**

**See excel sheet**
